# Validation of deep amplicon sequencing of *Dicrocoelium* in small ruminants from Northern regions of Pakistan

**DOI:** 10.1371/journal.pone.0302455

**Published:** 2024-04-29

**Authors:** Muhammad Asim Khan, Kiran Afshan, Sabika Firasat, Muhammad Abbas, Neil D. Sargison, Martha Betson, Umer Chaudhry

**Affiliations:** 1 Faculty of Biological Sciences, Department of Zoology, Quaid-i-Azam University, Islamabad, Pakistan; 2 Department of Comparative Biomedical Sciences, School of Veterinary Medicine, University of Surrey, Guildford, United Kingdom; 3 Royal (Dick) School of Veterinary Studies, University of Edinburgh, Edinburgh, United Kingdom; 4 School of Veterinary Medicine, St. George’s University True Blue, St. George’s Grenada, Caribbean; Universidade Federal de Minas Gerais, BRAZIL

## Abstract

*Dicrocoelium* lancet flukes cause significant production loss in ruminant livestock. Although co-infection with multiple *Dicrocoelium* species within a host is common, techniques for studying the composition of these complex parasite communities are lacking. The pathogenicity, epidemiology, and therapeutic susceptibility of different helminth species vary, and little is known about the interactions that take place between co-infecting species and their hosts. Here, we describe the first applicationof metabarcoding deep amplicon sequencing method to studythe *Dicrocoelium* species in sheep and goats. First, rDNA ITS-2 sequences of four *Dicrocoelium* species (*Dicrocoelium dendriticum*, *Dicrocoelium hospes*, *Dicrocoelium orientalis*, and *Dicrocoelium chinensis*) were extracted from the NCBI public database. Phylogenetic analysis revealed separate clades of *Dicrocoelium* species; hence, molecular differentiation between each species is possible in co-infections. Second, 202 flukes belonging to seventeen host populations (morphologically verified as belonging to the *Dicrocoelium* genus) were evaluated to determine the deep amplicon sequencing read threshold of an individual fluke for each of the four species. The accuracy of the method in proportional quantification of samples collected from single hosts was further assessed. Overall, 198 (98.01%) flukes were confirmed as *D*. *dendriticum* and 1.98% produced no reads. The comparison of genetic distances between rDNA ITS-2 revealed 86% to 98% identity between the *Dicrocoelium* species. Phylogenetic analysis demonstrated a distinct clustering of species, apart from *D*. *orientalis* and *D*. *chinensis*, which sit very close to each other in a single large clade whereas *D*. *hospes* and *D*. *dendriticum* are separated into their own clade. In conclusion each sample was identified as *D*. *dendriticum* based on the proportion of MiSeq reads and validated the presence of this group of parasites in the Gilgit Baltistan and Khyber Pakhtunkhwa provinces of Pakistan. The metabarcoding deep amplicon sequencing technology and bioinformatics pathway have several potential applications, including species interactions during co-infections, identifying the host and geographical distribution of *Dicrocoelium* in livestock, drug therapy response evaluation and understanding of the emergence and spread of drug resistance.

## Introduction

Dicrocoeliid liver flukes can infect the bile ducts of various domesticated and wild mammals around the globe [1–3]. Four species of the genus *Dicrocoelium*, namely *Dicrocoelium dendriticum*, *Dicrocoelium hospes*, *Dicrocoelium orientalis* and *Dicrocoelium chinensis* have been described as causes of dicrocoeliosis in domestic and wild ruminants [4]. It is an important trematode parasitic disease, causing significant production loss in pastoral livestock. Among those, *D*. *dendriticum* has been reported in Europe, Asia, northern Africa, and North America; *D*. *hospes* is endemic in sub-Saharan and west Africa; *D*. *chinensis* in Eastern Asia and Europe; and *D*. *orientalis* from the Baikal region of the former Soviet Union [5]. *Dicrocoelium* was initially identified in the Himalayan ranges of Pakistan [6], where it was found in 8.66% of liver samples and 3.93% were positive for *IgG* antibodies against *Dicrocoelium* [7], although earlier there were only anecdotal and unsubstantiated reports from Pakistan [8].

Although *Dicrocoelium* is frequently observed in ruminants, it also affects rabbits, pigs, dogs, horses, and humans [9]. This disease in humans has been linked to diarrhoea, flatulence, biliary obstruction, cholangitis, acute urticaria, and cirrhosis [10–12]. *Dicrocoelium* has a complex life cycle consisting of three hosts, including herbivores as definitive hosts and terrestrial snails and formicide ants as intermediate hosts [13,14]. Due to the involvement of two intermediate hosts, *Dicrocoelium* is highly affected by climatic and geographic factors. Temperature and humidity affected the survival of miracidia-containing eggs as well as the development of snails and ants in their respective environmental niches [7,15].

The faecal sedimentation method was frequently employed as a confirming test for *Dicrocoelium* infection in live animals [16]. These conventional methods lack the precision to accurately identify *Dicrocoelium* infection to the species level required to study the epidemiology, and ecology of dicrocoeliosis in specific regions. Next-generation genomic resources have potential applications in diagnosis, surveillance, treating and controlling parasitic diseases [17,18]. The sensitivity and specificity of molecular approaches have improved in recent years. Still, the reliability of infection reports varies considerably between affected areas because of the different molecular-based methods used [19]. Traditional PCR and Sanger sequencing methods amplify fragments of nuclear ribosomal genes, and their internal transcribed spacers have been developed for dicrocoeliid parasites [6]. The methods are helpful for accurately detecting *Dicrocoelium* spp., but heavily depend on sensitive and specific primers and have limitations regarding scalability and detection of co-infections [17]. A universal test to detect all *Dicrocoelium* spp. with equal reliability is needed to resolve these issues and improve surveillance systems. A high throughput deep amplicon sequencing using the Illumina Mi-Seq platform has the potential to open new areas of research to improve surveillance of separate *Dicrocoelium* species.

Morphology alone may not be enough to identify many digenean species. Morphological factors that make the differentiation of digenean species difficult include: the small size of adult stages and a scarcity of taxonomic characters, uncertainty about the validity of these traits [20]; cryptic species and phenotypic plasticity [21]. These factors can cause parasite diversity to be underestimated or exaggerated. Thus, molecular approaches, particularly primary sequence comparisons, are utilized to study life cycles, putative cryptic species, species complexes and their phylogeographical genetic structure, and phylogenetic research. The ITS-2 region of the rDNA cistron was chosen as the sequence target due to it having the appropriate level of species-specific variation for reliable species discrimination [22]. Here, we describe the development of a deep sequencing assay of the rDNA ITS-2 and validate its use to accurately quantify the species composition of *Dicrocoelium* communities present in field samples. We demonstrate that the approach is extremely robust and quantitatively accurate when applied to field populations of *Dicrocoelium* isolated from sheep and goats and highlight how this method can be used to investigate the biodiversity of various parasite populations in animal hosts.

## Materials and methods

### Study area and field samples

A cross-sectional sampling of *Dicrocoelium* in livestock was conducted from 14 known endemic regions of Pakistan’s Khyber Pakhtunkhwa and Gilgit Baltistan provinces [7,14]. Gilgit Baltistan borders China via the Khunjrab Pass, having an annual rainfall of 120 to 240 mm. Khyber Pakhtunkhwa shares a western border with Afghanistan and receives 124 mm of rain per year.

The flukes were obtained from slaughterhouses where animals were slain for various purposes in order to supply the population’s protein demands. The Bio-Ethical Committee (BEC) of Quaid-i-Azam University, Islambad provided ethical permission (Ref. No. #BEC-FBS-QAU2017). The samples were collected from March-September 2019, 2020 and 2021 [6,14]. The sample collection was carried out in local abattoirs in their residing area, where there is a known prevalence of dicrocoeliosis [13]. Twelve (sheep = 9 and goat = 3) livers were collected from ten abattoirs (Booni, Torkhow, Mastuj, Laspoor Valley, Brun, Gabral, Boyun, Chinar, Gasht and Chashma) in Khyber Pakhtunkhwa and five (sheep = 4 and goats = 1) livers from four abattoirs (Dalomal, Yasin Valley, Raushan and Chalt Nagar) in Gilgit Baltistan province. One hundred forty-four individual flukes from Khyber Pakhtunkhwa and 58 from Gilgit Baltistan were collected in total. The flukes collected from each liver were referred to as a single population (S1 Table). The livers were transported on ice to the laboratory, where flukes were extracted from the bile ducts. The flukes were cleaned with phosphate-buffered saline (PBS) to remove any adhering material before being preserved in 70% ethanol for morphometric and DNA analysis.

### Morphological characteristics of adult flukes

A total of 202 flukes were selected for morphological characterisation. The flukes were fixed between two glass slides in formalin-acetic acid alcohol solution, stained with hematoxylin (Sigma-Aldrich) and mounted in Canada balsam. Each fluke was morphologically identified using standardised measurements, including the orientation of the testes, followed by twenty-six morphometric characters [9,23,24]. Eggs were isolated from the uterus of adult lancet flukes. The measurements were made with a microscope (Leica LB Germany) and images were captured by a Canon digital camera (Japan).

### Genomic DNA isolation, metabarcoded PCR amplification, and Illumina Mi-Seq run

A small tissue piece of approximately 1 mg was taken from the head of each *Dicrocoelium* fluke (in a total of 202) to avoid contamination by fertilized eggs. Each tissue sample was rinsed for 5 min in a petri dish with distilled water before being lysed in 25 μl worm-lysis solution (Viagen) using a protocol previously described by Rehman et al. [25,26] (S2 Table). DNA lysates were stored at -80°C until required.

Metabarcoded PCR amplification was applied to the individual worms for species identification using the rDNA ITS-2 marker. A 337 bp fragment of ITS-2 rDNA was amplified from each of the 202 individual *Dicrocoelium* flukes from 17 populations (S1 Table). The modified primer sets, adapter and barcoded PCR amplification, and magnetic bead purification were previously described [25,26]. Subsequently, 10 μl of each barcoded PCR product of the rDNA ITS-2 locus was combined to make a pooled library and run on agarose gel electrophoresis to separate PCR products. The products were excised from the gel using commercial kits (QIAquick Gel Extraction Kit, Qiagen, Germany) and 20 μl of eluted DNA was then purified using AMPure XP Magnetic Beads (1X) (Beckman Coulter, Inc.) to form a single purified DNA pooled library. The library was measured with KAPA qPCR library quantification kit (KAPA Biosystems, USA) and then run on an Illumina MiSeq Sequencer using a 500-cycle pair-end reagent kit (MiSeq Reagent Kits v2, MS-103-2003) at a concentration of 15nM with the addition of 15% Phix Control v3 (Illumina, FC-11-2003).

### Bioinformatics data analysis

The FASTQ data was received in a.tar file, which was extracted using the "tar -xaf" command, followed by unzipping the file with the "gzip" command and FASTQ files were retrieved using "*.gz -exec gunzip" command. Post-run processing separated the sequences according to the recognized barcoded indices and generated FASTQ files (freely available through the Mendeley database DOI: 10.17632/9knwyjtrkx.1). The Mi-Seq data analysis was performed with a bespoke pipeline using Mothur v1.39.5 software [27,28] with modifications in the standard operating procedures of Illumina Mi-Seq [25,26] (freely available through the Mendeley database DOI: 10.17632/9knwyjtrkx.1).

The raw paired-end reads were analysed to combine the two sets of reads for each parasite population using make.contigs command, which requires ‘stability.files’ as an input. The ‘make.contigs’ command extracts sequence quality score data from FASTQ files, creating complements of the reverse and forward reads and joins them into contigs. It aligns the pairs of sequence reads and compares the alignments to identify any positions where the two reads disagree. Next, there was a need to remove any sequences with ambiguous bases using the ‘screen.seqs’ command. The above dataset was aligned with *Dicrocoelium* rDNA ITS-2 consensus sequences (generated in section 2.5.) and reference taxonomy libraries (freely available through the Mendeley database DOI: 10.17632/9knwyjtrkx.1) created from the NCBI database where the sequences start and end with the primer sets. Hundreds of thousands of rDNA ITS-2 reads were generated from the dataset of individual flukes using classify.seqs command. The rDNA ITS-2 classified data of 202 individual *Dicrocoelium* flukes were analysed in Microsoft Excel v16.35 to display the presence of *Dicrocoelium* species in corresponding hosts based on the rDNA ITS-2 reads (S3 Table). Finally, the "get.lineage" command was utilized to retrieve the align file containing 84408 *Dicrocoelium* sequences, which was subjected to the "unique.seqs" command, producing 11910 unique sequences.

The FASTA file of the most repeated amplicon sequence variants-ASVs (one of the inferred single DNA sequences recovered from a high-throughput analysis of genetic marker) from 11910 unique sequences was generated in Bioconductor (version 3.17) installed in RStudio v1.2.5033 software [29]. The FASTA file was trimmed and aligned using the MUSCLE tool of Geneious v10.2.5 (Biomatters Ltd, New Zealand) with a consensus sequence library of four NCBI-extracted *Dicrocoelium* species (*D*. *orientalis*, *D*. *hospes*, *D*. *dendriticum* and *D*. *chinensis*). This enabled the presence of *Dicrocoelium* species in corresponding field samples based on the rDNA ITS-2 ASVs to be determined. Finally, a phylogenetic tree of the rDNA ITS-2 ASVs along with the NCBI-extracted *Dicrocoelium* species was constructed by gamma-distributed Kimura 2-parameter (K2+G) model using the Maximum Likelihood method in the MEGA X software, with a bootstrap value of 1000 [30,31].

#### Consensus sequence library preparation and the analysis of rDNA ITS-2 NCBI GenBank sequences of common liver flukes

The consensus sequence library was developed using filtered sequence reads of four *Dicrocoelium* (*D*. *orientalis*, *D*. *hospes*, *D*. *dendriticum* and *D*. *chinensis*) and two *Fasciola* (*F*. *gigantica*, *F*. *hepatica*) species extracted from the NCBI GenBank to account for genetic variations (for more detail, see Mendeley database at DOI: 10.17632/9knwyjtrkx.1). The obtained rDNA ITS-2 filtered consensus sequence were aligned using the MUSCLE alignment tool of Geneious v10.2.5 (Biomatters Ltd, New Zealand). A phylogenetic tree of the rDNA ITS-2 consensus sequences of the four *Dicrocoelium* and two *Fasciola* species was constructed by gamma-distributed Kimura 2-parameter (K2+G) model using the maximum likelihood method in the MEGA X software, with a bootstrap value of 1000 [30,31]. The genetic distances between ITS-2 rDNA sequences of four *Dicrocoelium* species were computed using Geneious v10.2.5 and are shown in percentage similarity.

## Results

### *Dicrocoelium* species confirmation in field samples based on morphological characteristics

**Fig 1:**
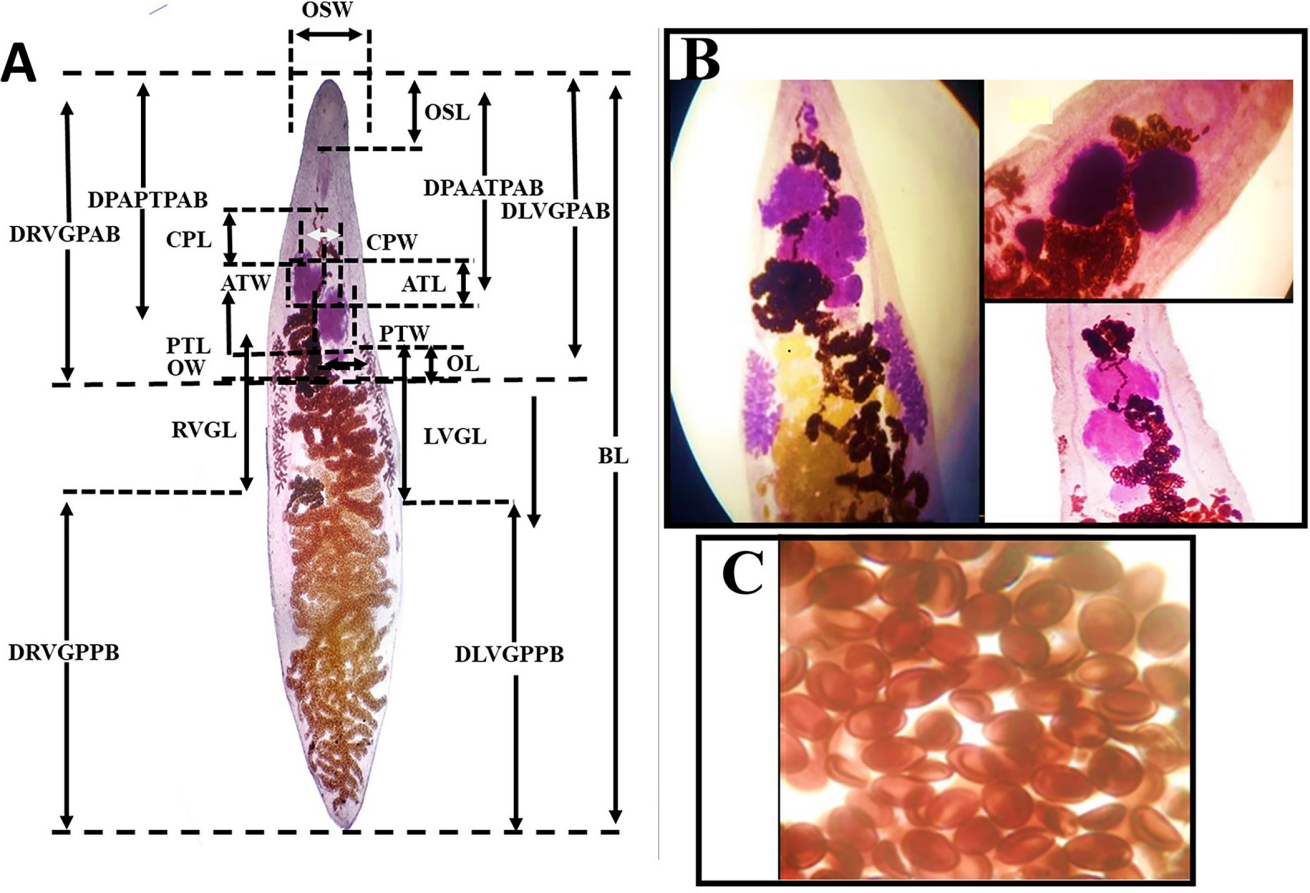
(A) *Dicrocoelium dendriticum* worm showing morphometric features used as variables. [B) Hematoxylin-stained *D*. *dendriticum*, variation in testis shape, ovary, and vitelline glands (C) Uterine eggs of *D*. *dendriticum*.

A total of 202 individual *Dicrocoelium*, comprising 17 fluke populations from Khyber Pakhtunkhwa and Gilgit Baltistan provinces, were analysed based on morphological traits. All samples were identified as *D*. *dendriticum* based on shape and size (Table 1, Fig 1). *Dicrocoelium dendriticum* has a translucent, dorsoventrally flattened body that is between 1.6 and 8 mm long and 0.48 and 1.84 mm wide. The oral sucker of *D*. *dendriticum* is subterminal and measures 80–400 μm. The slightly larger ventral sucker (80–408 μm) is in the anterior quarter of the body (Table 1). Just behind the intestinal bifurcation, the genital pore is located (Fig 1). The ovary is beneath the posterior testis and measures 40–320 μm by 40–480 μm (Table 1). The anterior end of the slightly lobed testes is close to the posterior margin of the ventral sucker and to arrange in the body in an oblique manner (Fig 1). The anterior testis is 88–640 μm long and 92–736 μm wide, while the posterior testis is 88–728 x 120–896 μm (Table 1). At the level of ventral sucker, the vasa efferentia unite to form vas deferens which enters the cirrus pouch and forms a seminal vesicle (Fig 1). The operculate eggs have a diameter of 12 x 8 μm indicating the *D*. *dendriticum* species (Fig 1).

**Table 1:** Morphometric values of *Dicrocoelium dendriticum* field samples (n = 202).

Measurement Parameters	Mean ± SD (mm)	Min–Max (mm)
Body length (BL)	3.75 ± 1.55	1.6–8
Maximal body width (BW)	0.94 ± 0.33	0.48–1.84
Oral sucker length (OSL)	175.34 ± 75.53	80–400
Oral sucker width (OSW)	168.26 ± 70.31	80–328
Ventral sucker length (VSL)	194.68 ± 80.08	80–408
Ventral sucker width (VSW)	191.48 ± 76.84	80–404
Cirrus pouch length (CPL)	137.6 ± 62.43	80–328
Cirrus pouch width (CPW)	70.83 ± 31.09	40–252
Anterior testes length (ATL)	265.18 ± 115.26	88–640
Anterior testes width (ATW)	299.73 ± 140.59	92–736
Posterior testes length (PTL)	290.23 ± 141.93	88–728
Posterior testes width (PTW)	310.83 ± 148.92	120–896
Right vitelline length (RVL)	877.73 ± 415.72	404–2172
Right vitelline width (RVW)	160.53 ± 76.99	40–408
Left vitelline length (LVL)	862.85 ± 397.47	400–2120
Left vitelline width (LVW)	166.15 ± 76.55	40–400
Ovary length (OL)	117.38 ± 65.63	40–320
Ovary width (OW)	167.45 ± 99.6	40–480
Egg length (EL)	12 ± 0	12–12
Egg width (EW)	8 ± 0	8–8
Distance from front of the anterior testes to front of the body (DPAATPAB)	757.73 ± 335.63	304–1880
Distance from posterior part of the testes to front of the body (DPAPTPAB)	941.3 ± 387.65	320–2400
Distance from the right vitelline gland to the front of the body (DRVGPAB)	1327.5 ± 528.48	648–3040
Distance from the right vitelline the tail of the body (DRVGPPB)	1500.83 ± 702.05	560–3612
Distance from the right vitelline to the tail of the body (DLVGPAP)	1347.55 ± 516.78	648–2880
Distance from the left vitelline to the front of the body (DLVGPPB)	1580.7 ± 746.21	568–3696

Min: minimum, Max: Maximum; SD: Standard deviation; mm: Millimeters.

### Assessment of rDNA ITS-2 genetic variations in NCBI sequence data

In total, 87 rDNA ITS-2 consensus sequences were obtained representing four *Dicrocoelium* species (*D*. *orientalis = 4*, *D*. *hospes = 2*, *D*. *dendriticum = 61* and *D*. *chinensis = 20*) and 27 representing two *Fasciola* species (*F*. *gigantica* = 14, *F*. *hepatica* = 13) (Supplementary Mendeley database at DOI: 10.17632/9knwyjtrkx.1). The comparison of genetic distances between rDNA ITS-2 revealed 86% to 98% identity between the *Dicrocoelium* species (Table 2). The most closely-related species of *Dicrocoelium* (*D*. *orientalis* and *D*. *chinensis*) could still be reliably differentiated by virtue of rDNA ITS-2 sequence variations (98%). In addition to confirming the species- specific identity, these rDNA ITS-2 sequences identified three sites that showed 0.8% intraspecific variation for *D*. *orientalis* and *D*. *hospes*, nine sites that showed 2.67% intraspecific variation for *D*. *dendriticum*, and twelve sites that showed 3.56% intraspecific variation for *D*. *chinensis* (for more information Supplementary Mendeley database Genebank sequences file). Similarly, the comparison of genetic distances between rDNA ITS-2 revealed 98% identity between the *Fasciola* species (data not shown). Overall, there are consistent genetic variations in the *Dicrocoelium* and *Fasciola* species between the rDNA ITS-2 variants.

**Table 2:** The genetic distances between rDNA ITS-2 sequences of four *Dicrocoelium* species.

	*D*. *orientalis*	*D*. *hospes*	*D*. *dendriticum*
***D*. *hospes***	87%		
***D*. *dendriticum***	95%	86%	
***D*. *chinensis***	98%	86%	95%

### Phylogenetic analysis of rDNA ITS-2 identified in the NCBI sequence data

**Fig 2:**
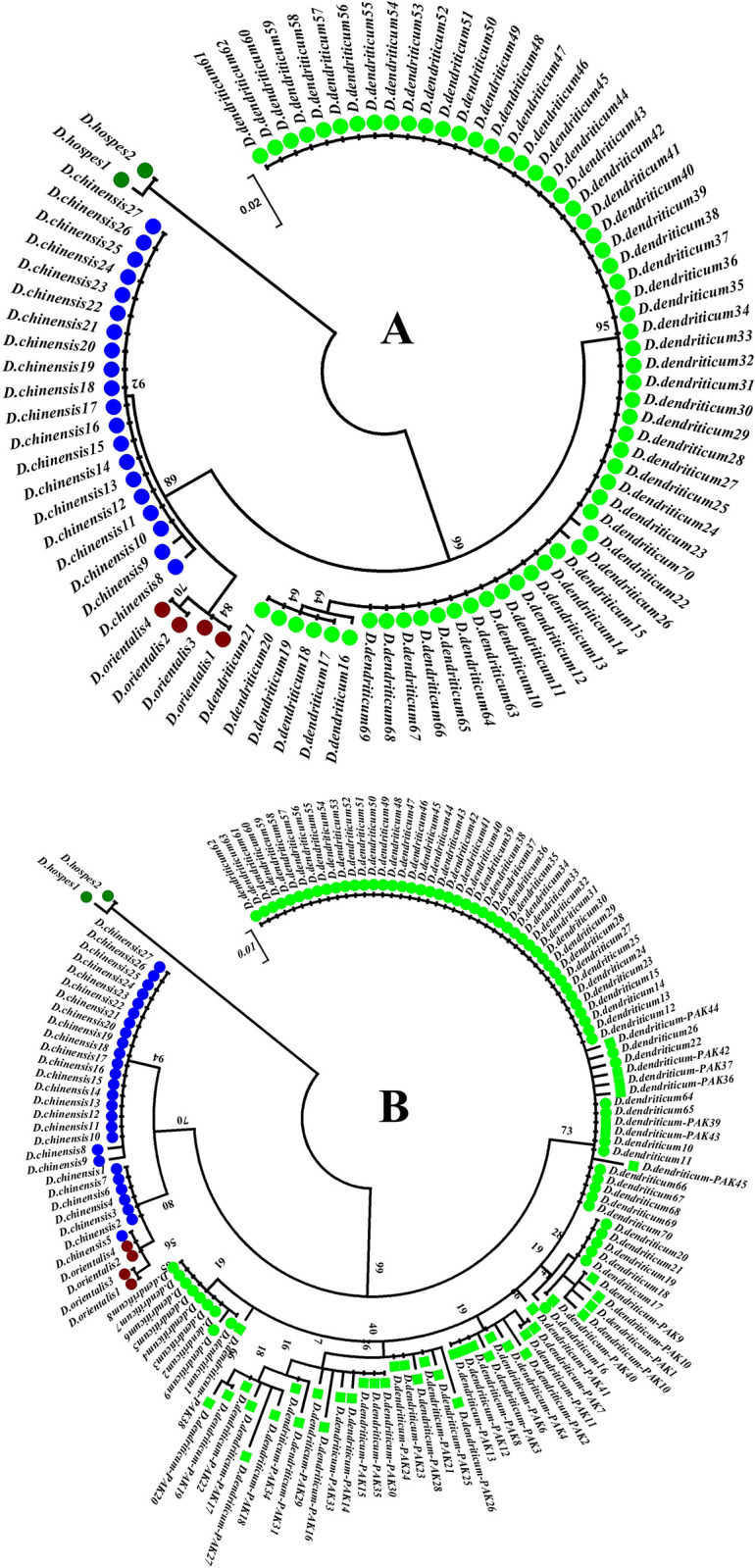
(A) Maximum-likelihood tree of 87 rDNA ITS-2 sequences from four *Dicrocoelium* species (*D*. *orientalis*, *D*. *hospes*, *D*. *dendriticum* and *D*. *chinensis*) obtained from NCBI Genbank. (B) Maximum-likelihood tree of 44 rDNA ITS-2 ASVs obtained from *D*. *dendriticum* field and 87 rDNA ITS-2 sequences from four *Dicrocoelium* species (*D*. *orientalis*, *D*. *hospes*, *D*. *dendriticum* and *D*. *chinensis*) obtained from NCBI Genbank. Each species is indicated with different coloured dots.

A maximum-likelihood tree was constructed from consensus sequences (87 *Dicrocoelium*, and 27 *Fasciola*) of rDNA ITS-2 locus extracted from the NCBI Genbank (Figs 2A and S1). This demonstrated a distinct clustering of species, apart from *D*. *orientalis* and *D*. *chinensis*, which sit very close to each other in a single large clade whereas *D*. *hospes* and *D*. *dendriticum* are separated into their own clade (Fig 2A). Similarly, distinct clustering of rDNA ITS-2 loci of *Dicrocoelium* and *Fasciola* was well-supported for each species (S1 Fig). The topology of both trees (Figs 2 and S1) was confirmed by high bootstrap confidence intervals, ranging from 64–99 (Figs 2A and S1).

#### *Dicrocoelium* species confirmation in field samples based on the deep amplicon sequencing

rDNA ITS-2 amplicons from 202 individual *Dicrocoelium*, comprising 17 fluke populations from Khyber Pakhtunkhwa and Gilgit Baltistan provinces, were run on the Illumina Mi-Seq platform (Table 3 and S3 Table). Overall, 198 (98.01%) out of 202 flukes collected from 13 sheep and 4 goat populations were confirmed as *D*. *dendriticum* (Table 3) and 4 (1.98%) flukes produced no reads. Among the Khyber Pakhtunkhwa province, 143 (70.79%) out of 144 fluke*s* collected from 9 sheep and 3 goat populations were confirmed as *D*. *dendriticum*, and 1 (0.49%) produced no reads. Among the Gilgit Baltistan province, 55 (27.22%) out of 58 fluke*s* collected from 4 sheep and one goat populations were confirmed as *D*. *dendriticum*, and 3 (1.48%) produced no reads (Table 3). No other *Dicrocoelium* species were identified in the field samples.

**Table 3:** *Dicrocoelium* species identification of individual flukes from 17 populations based on deep sequencing of rDNA ITS-2 genetic marker. Each population represents the total flukes collected from an individual host.

Population	Area	Province	Host	Total Flukes	*D*. *dendriticum*	*D*. *chinensis*	*D*. *orientalis*	*D*. *hospe*s	No reads
P1	Booni	Khyber Pakhtunkhwa	Sheep	13	13	-	-	-	-
P2	Booni	Khyber Pakhtunkhwa	Sheep	12	12	-	-	-	-
P3	Booni	Khyber Pakhtunkhwa	Sheep	12	12	-	-	-	-
P4	Torkhow	Khyber Pakhtunkhwa	Sheep	12	12	-	-	-	-
P5	Mastuj	Khyber Pakhtunkhwa	Sheep	13	13	-	-	-	-
P6	Laspoor Valley	Khyber Pakhtunkhwa	Sheep	12	12	-	-	-	-
P7	Brun	Khyber Pakhtunkhwa	Sheep	12	12	-	-	-	-
P12	Gabral	Khyber Pakhtunkhwa	Sheep	12	12	-	-	-	-
P13	Boyun	Khyber Pakhtunkhwa	Sheep	12	11	-	-	-	1
P14	Chinar	Khyber Pakhtunkhwa	Goat	12	12	-	-	-	-
P15	Gasht	Khyber Pakhtunkhwa	Goat	12	12	-	-	-	-
P17	Chashma	Khyber Pakhtunkhwa	Goat	10	10	-	-	-	-
Total				144 (71.28%)	143 (70.79%)				1 (0.49%)
P8	Dalomal	Gilgit Baltistan	Sheep	12	12	-	-	-	-
P9	Yasin Valley	Gilgit Baltistan	Sheep	12	12	-	-	-	-
P10	Raushan	Gilgit Baltistan	Sheep	12	12	-	-	-	-
P11	Raushan	Gilgit Baltistan	Sheep	10	10	-	-	-	-
P16	Chalt Nagar	Gilgit Baltistan	Goat	12	9	-	-	-	3
Total				58 (28.71%)	55 (27.22%)				3 (1.48%)
**Total**				202 (100%)	198 (98.01%)	0 (0%)	0 (0%)	0 (0%)	4 (1.98%)

### Phylogenetic analysis of *Dicrocoelium* identified in field samples

In total, 44 rDNA ITS-2 ASVs were identified among *Dicrocoelium* sequences present in the field samples. A maximum-likelihood tree was constructed from 44 ITS-2 ASVs from *Dicrocoelium* field samples and 87 *Dicrocoelium* consensus sequences from NCBI Genbank (*D*. *orientalis = 4*, *D*. *hospes = 2*, *D*. *dendriticum = 61* and *D*. *chinensis = 20*) (Fig 2B). Overall, the data shows that the *Dicrocoelium* species separated into different clades (Fig 2B). All field samples were found in the *D*. *dendriticum* clade Overall, the data shows that the *Dicrocoelium* species separated into different clades (Fig 2B). *Dicrocoelium dendriticum* data comprising of both field ASVs and the NCBI Genbank sequences show all field samples sitting with their respective clades of Genbank sequences. The *D*. *orientalis* and *D*. *chinensis* sit very close to each other in a single large clade and *D*. *hospes* are separated into their own clade (Fig 2B). The comparison of genetic distances between rDNA ITS-2 revealed similar interspecific differenation between the *Dicrocoelium* species (Table 2). In addition to confirming the species-specific identity, these rDNA ITS-2 sequences identified nine sites that showed 2.67% intraspecific variation for *D*. *dendriticum* NCBI GeneBank sequences, but seven sites that showed 2.07% intraspecific variations for *D*. *dendriticum* field ASVs (for more information Supplementary Mendeley database Genebank sequences and ASVs files).

### Discussion

Phenotypic traits such as body width and length, are conventionally used to identify adult flukes by their species [32]. In present study *D*. *dendriticum* was morphologically identified based on the testes orientation, overall size, and level of maximum body width as described by Otranto [9]. However, morphological differences may be skewed due to the existence of intermediate forms [33]. Furthermore, advance approaches are required for accurate species idetitity as morphologically intraspecific variation was greater than interspecific variation between different fluke species and specimens from various hosts [34].

High throughput deep amplicon sequencing using the Illumina Mi-Seq platform is relatively low-cost and potentially less error-prone [35]. The method has transformed the study of veterinary and human haemoprotozoa [17–19], and clade V parasitic nematodes [36–38], and has the potential to improve surveillance of *Dicrocoelium* species as previously demonstrated by the concept of a ‘tremobiome’ for the quantification of the *Fasciola* and *Calicophoron* species [25,26,39].

This approach targets genetic variations within defined regions of the trematode genome to detect and quantify any species belonging to the parasite of interest. *Dicrocoelium* rDNA ITS-2 is a suitable genetic target due to a genome-wide distribution and high copy number of species-specific variable sequences flanked by highly conserved sequences to enable universal primer binding and discrimination between trematode species [25,26,39]. Use of primers binding to conserved sites and analysis of up to 600 bp sequence reads allows *Dicrocoelium* species to be detected. Because barcoded primers allow multiple samples to be pooled and sequenced in a single Mi-Seq run, the technology is well suited for high-throughput analysis. By multiplexing the barcoded primer combinations, it is possible to run 384 samples at once on a single Illumina Mi-Seq flow cell, helping to reduce the cost [35].

Here, we first analysed rDNA ITS-2 sequences of four *Dicrocoelium* species (*D*. *orientalis*, *D*. *hospes*, *D*. *dendriticum* and *D*. *chinensis*) and two *Fasciola* (*F*. *gigantica*, *F*. *hepatica*) from the NCBI public database, showing consistent genetic variations between species. Comparison of genetic distances between rDNA ITS-2 revealed genetic variations between the *Dicrocoelium* and *Fasciola* species, allowing practical differentiation of the Dicrocoeliidae and Fasciolidae family. The maximum-likelihood tree shows separate clades of *Dicrocoelium* (*D*. *orientalis*, *D*. *hospes*, *D*. *dendriticum* and *D*. *chinensis*) and *Fasciola* (*F*. *gigantica*, *F*. *hepatica*) species; hence, molecular differentiation between each species is possible in co-infections. The PCR based on mtDNA cox1 could efficiently differentiate *D*. *dendriticum* and *D*. *chinensis* [40]. However, low-level sequencing variations in mtDNA cox1, nad1, and cytb were discovered in *D*. *dendriticum* samples from various locations in Shaanxi Province, northern China [41]. In another study, the cox1 and nad1 fragments were used to reveal the intra-population genetic variations of D. chinensis from domestic yaks in Gansu and Sichuan Provinces, revealing lower intra-population genetic variations (1%), but higher inter-species differences (>10%) among common trematodes [42]. Phylogenetic comparison of published *D*. *dendriticum* cytochrome oxidase-1 (COX-1) mitochondrial DNA sequences with those from *D*. *chinensis* was assessed and 4 unique haplotypes were confirmed [6].

Secondly, we use high throughput amplicon sequencing followed by morphological confirmation of the species-specific infections of *D*. *dendriticum* in sheep and goats in Pakistan’s Khyber Pakhtunkhwa and Gilgit Baltistan provinces. Dicrocoeliid liver flukes have previously been reported in India [43], China [44,45], Iran [13], Canada [5], France [16], Germany [46] and Italy [47]. In a small-scale study, dicrocoeliosis was recently reported in Pakistan’s Khyber Pakhtunkhwa and Gilgit Baltistan provinces [6]. Our confirmation of *D*. *dendriticum* in Pakistan highlights the need to better understand parasite’s biology, such as identifying the species of snail and ant that may act as competent intermediate hosts. Identifying *D*. *dendriticum* has implications for diagnosing and controlling dicrocoeliosis in Pakistan. These findings show the potential for the development of population genetics tools to study the epidemiology of this parasite, potentially arising as a consequence of changing management and climatic conditions. A better understanding of the molecular evolutionary biology and phylogenetics of *D*. *dendriticum* will help inform novel fluke control methods that are now needed.

In summary, we used the metabarcoding deep amplicon sequencing using an Illumina Mi-Seq platform to reliably describe the *Dicrocoelium* species in liver samples collected from sheep and goats. Our results are proof of concept for using this method in disease surveillance programmes in farm animals in the resource-poor setting of the endemic regions with through increasing laboratory capacity. The application of this approach is not *Dicrocoelium*-specific and could have future applications for assessing other animals and trematode species. This technology has practical applications in monitoring multiplicity of infections, the geographical distribution of parasites and co-infection described in other trematode parasites [25,26,39], and changes in parasite diversity after the emergence and spread of drug resistance.

## Supporting information

S1 Fig Maximum-likelihood tree of rDNA ITS-2 sequences from four *Dicrocoelium* species (*D*. *orientalis*, *D*. *hospes*, *D*. *dendriticum* and *D*. *chinensis*) and two *Fasciola* species (*F*. *gigantica* and *F*. *hepatica*) obtained from NCBI Genbank. The sequences were first aligned using the MUSCLE tool of the Geneious v9.0.1 software. The neighbour-joining algorithm (Kimura 2+G parameter model) was computed with 1000 bootstrap replicates using MEGA5 software created by Biomatters. Each species is identicated with different coloured dots.
(JPG)

S1 Table The samples were collected during the peak *Dicrocoelium* transmission seasons from Khyber Pakhtunkhwa and Gilgit Baltistan provinces of Pakistan. (DOCX)

S2 Table rDNA ITS-2 primer sequences for the amplification of Dicrocoelium. Forward and reverse primer sets are underlined, N’s are bolded, and adapters are in italic format.
(DOCX)

S3 Table Deep amplicon sequencing data of 202 individual *Dicrocoelium*, comprising 17 fluke populations from Khyber Pakhtunkhwa and Gilgit Baltistan provinces of Pakistan. (DOCX)
