# Supplementary figures and images for: Validation of deep amplicon sequencing of Dicrocoelium in small ruminants from Northern regions of Pakistan
Source: PLoS One. 2024 Apr 29;19(4):e0302455. doi: 10.1371/journal.pone.0302455 (PMC11057770; doi:10.1371/journal.pone.0302455)

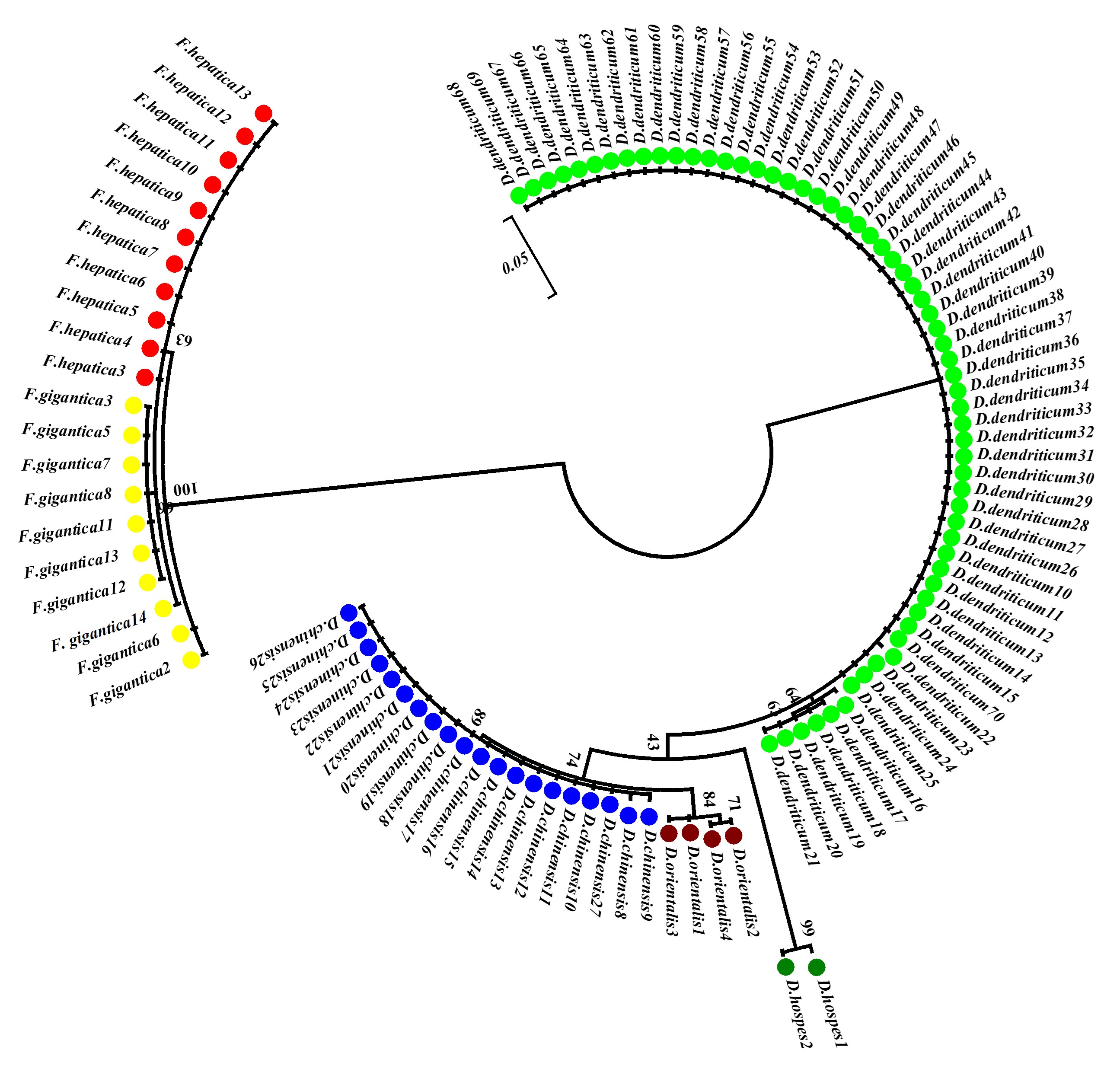

Supplement: S1 Fig — The sequences were first aligned using the MUSCLE tool of the Geneious v9.0.1 software. The neighbour-joining algorithm (Kimura 2+G parameter model) was computed with 1000 bootstrap replicates using MEGA5 software created by Biomatters. Each species is identicated with different coloured dots. (JPG) [file pone.0302455.s001.jpg]
