# Supplementary material for: Validation of deep amplicon sequencing of Dicrocoelium in small ruminants from Northern regions of Pakistan
Source: PLoS One. 2024 Apr 29;19(4):e0302455. doi: 10.1371/journal.pone.0302455 (PMC11057770; doi:10.1371/journal.pone.0302455)
Supplement: S1 Table — (DOCX) [file pone.0302455.s002.docx]

| **Population** | **Flukes** | **Host** | **Area** | **Province** |
| --- | --- | --- | --- | --- |
| P1 | 13 | Sheep | Booni | Khyber Pakhtunkhwa |
| P2 | 12 | Sheep | Booni | Khyber Pakhtunkhwa |
| P3 | 12 | Sheep | Booni | Khyber Pakhtunkhwa |
| P4 | 12 | Sheep | Torkhow | Khyber Pakhtunkhwa |
| P5 | 13 | Sheep | Mastuj | Khyber Pakhtunkhwa |
| P6 | 12 | Sheep | Laspoor Valley | Khyber Pakhtunkhwa |
| P7 | 12 | Sheep | Brun | Khyber Pakhtunkhwa |
| P8 | 12 | Sheep | Dalomal | Gilgit Baltistan |
| P9 | 12 | Sheep | Yasin Valley | Gilgit Baltistan |
| P10 | 12 | Sheep | Raushan | Gilgit Baltistan |
| P11 | 10 | Sheep | Raushan | Gilgit Baltistan |
| P12 | 12 | Sheep | Gabral | Khyber Pakhtunkhwa |
| P13 | 12 | Sheep | Boyun | Khyber Pakhtunkhwa |
| P14 | 12 | Goat | Chinar | Khyber Pakhtunkhwa |
| P15 | 12 | Goat | Gasht | Khyber Pakhtunkhwa |
| P16 | 12 | Goat | Chalt Nagar | Gilgit Baltistan |
| P17 | 10 | Goat | Chashma | Khyber Pakhtunkhwa |

**Supplementary Table S1:** The samples were collected during the peak *Dicrocoelium* transmission seasons from Khyber Pakhtunkhwa and Gilgit Baltistan provinces of Pakistan
