# Supplementary material for: Validation of deep amplicon sequencing of Dicrocoelium in small ruminants from Northern regions of Pakistan
Source: PLoS One. 2024 Apr 29;19(4):e0302455. doi: 10.1371/journal.pone.0302455 (PMC11057770; doi:10.1371/journal.pone.0302455)
Supplement: S2 Table — Forward and reverse primer sets are underlined, N’s are bolded, and adapters are in italic format. (DOCX) [file pone.0302455.s003.docx]

**Supplementary Table S2**. rDNA ITS-2 primer sequences for the amplification of Dicrocoelium. Forward and reverse primer sets are underlined, N’s are bolded, and adapters are in italic format.

| **Sequences (5'-3')** | **Primer name** | **Target region** | **Direction** |
| --- | --- | --- | --- |
| *TCGTCGGCAGCGTCAGATGTGTATAAGAGACAG*GGTGGATCACTCGGCTCG*T*G | AD_For | rDNA ITS-2 | Forward |
| *TCGTCGGCAGCGTCAGATGTGTATAAGAGACAG***N**GGTGGATCACTCGGCTCG*T*G | AD_For-1N | rDNA ITS-2 | Forward |
| *TCGTCGGCAGCGTCAGATGTGTATAAGAGACAG***NN**GGTGGATCACTCGGCTCG*T*G | AD_For-2N | rDNA ITS-2 | Forward |
| *TCGTCGGCAGCGTCAGATGTGTATAAGAGACAG***NNN**GGTGGATCACTCGGCTCG*T*G | AD_For-3N | rDNA ITS-2 | Forward |
| *GTCTCGTGGGCTCGGAGATGTGTATAAGAGACAG*TTCCTCCGCTTAGTGATAT*G*C | AD_Rev | rDNA ITS-2 | Reverse |
| *GTCTCGTGGGCTCGGAGATGTGTATAAGAGACAG***N**TTCCTCCGCTTAGTGATAT*G*C | AD_Rev-1N | rDNA ITS-2 | Reverse |
| *GTCTCGTGGGCTCGGAGATGTGTATAAGAGACAG***NN**TTCCTCCGCTTAGTGATAT*G*C | AD_ Rev-2N | rDNA ITS-2 | Reverse |
| *GTCTCGTGGGCTCGGAGATGTGTATAAGAGACAG***NNN**TTCCTCCGCTTAGTGATAT*G*C | AD_ Rev-3N | rDNA ITS-2 | Reverse |
|  |  |  |  |
