# Supplementary material for: Validation of deep amplicon sequencing of Dicrocoelium in small ruminants from Northern regions of Pakistan
Source: PLoS One. 2024 Apr 29;19(4):e0302455. doi: 10.1371/journal.pone.0302455 (PMC11057770; doi:10.1371/journal.pone.0302455)
Supplement: S3 Table — (DOCX) [file pone.0302455.s004.docx]

**Supplementary Table S3.** Deep amplicon sequencing data of 202 individual *Dicrocoelium*, comprising 17 fluke populations from Khyber Pakhtunkhwa and Gilgit Baltistan provinces of Pakistan.

| **Sample** | **ITS2 *D.dendriticum* reads** | **ITS 2 *D. chinensis* reads** | | **ITS-2 *D. orientalis* reads** | | **ITS-2 *D. hospes* reads** | | **Host** | | **Population** | | **Area** | | | **Province** | |  |
| --- | --- | --- | --- | --- | --- | --- | --- | --- | --- | --- | --- | --- | --- | --- | --- | --- | --- |
| S1 | 1633 | | 0 | | 0 | | 0 | | Sheep | | 1 | | P 1 | Booni | | Khyber Pakhtunkhawa | |
| S2 | 1889 | | 0 | | 0 | | 0 | | Sheep | | 2 | |  | Booni | | Khyber Pakhtunkhawa | |
| S3 | 1269 | | 0 | | 0 | | 0 | | Sheep | | 3 | |  | Booni | | Khyber Pakhtunkhawa | |
| S4 | 1233 | | 0 | | 0 | | 0 | | Sheep | | 4 | |  | Booni | | Khyber Pakhtunkhawa | |
| S5 | 1012 | | 0 | | 0 | | 0 | | Sheep | | 5 | |  | Booni | | Khyber Pakhtunkhawa | |
| S6 | 1973 | | 0 | | 0 | | 0 | | Sheep | | 6 | |  | Booni | | Khyber Pakhtunkhawa | |
| S7 | 1580 | | 0 | | 0 | | 0 | | Sheep | | 7 | |  | Booni | | Khyber Pakhtunkhawa | |
| S8 | 1343 | | 0 | | 0 | | 0 | | Sheep | | 8 | |  | Booni | | Khyber Pakhtunkhawa | |
| S9 | 1497 | | 0 | | 0 | | 0 | | Sheep | | 9 | |  | Booni | | Khyber Pakhtunkhawa | |
| S10 | 1233 | | 0 | | 0 | | 0 | | Sheep | | 10 | |  | Booni | | Khyber Pakhtunkhawa | |
| S11 | 1837 | | 0 | | 0 | | 0 | | Sheep | | 11 | |  | Booni | | Khyber Pakhtunkhawa | |
| S12 | 1932 | | 0 | | 0 | | 0 | | Sheep | | 12 | |  | Booni | | Khyber Pakhtunkhawa | |
| S13 | 1804 | | 0 | | 0 | | 0 | | Sheep | | 13 | |  | Booni | | Khyber Pakhtunkhawa | |
| S14 | 1049 | | 0 | | 0 | | 0 | | Sheep | | 1 | | P 2 | Booni | | Khyber Pakhtunkhawa | |
| S15 | 1573 | | 0 | | 0 | | 0 | | Sheep | | 2 | |  | Booni | | Khyber Pakhtunkhawa | |
| S16 | 1567 | | 0 | | 0 | | 0 | | Sheep | | 3 | |  | Booni | | Khyber Pakhtunkhawa | |
| S17 | 1251 | | 0 | | 0 | | 0 | | Sheep | | 4 | |  | Booni | | Khyber Pakhtunkhawa | |
| S18 | 1840 | | 0 | | 0 | | 0 | | Sheep | | 5 | |  | Booni | | Khyber Pakhtunkhawa | |
| S19 | 1801 | | 0 | | 0 | | 0 | | Sheep | | 6 | |  | Booni | | Khyber Pakhtunkhawa | |
| S20 | 1962 | | 0 | | 0 | | 0 | | Sheep | | 7 | |  | Booni | | Khyber Pakhtunkhawa | |
| S21 | 1080 | | 0 | | 0 | | 0 | | Sheep | | 8 | |  | Booni | | Khyber Pakhtunkhawa | |
| S22 | 1829 | | 0 | | 0 | | 0 | | Sheep | | 9 | |  | Booni | | Khyber Pakhtunkhawa | |
| S23 | 1644 | | 0 | | 0 | | 0 | | Sheep | | 10 | |  | Booni | | Khyber Pakhtunkhawa | |
| S24 | 1328 | | 0 | | 0 | | 0 | | Sheep | | 11 | |  | Booni | | Khyber Pakhtunkhawa | |
| S25 | 1677 | | 0 | | 0 | | 0 | | Sheep | | 12 | |  | Booni | | Khyber Pakhtunkhawa | |
| S26 | 1480 | | 0 | | 0 | | 0 | | Sheep | | 1 | | P 3 | Booni | | Khyber Pakhtunkhawa | |
| S27 | 1714 | | 0 | | 0 | | 0 | | Sheep | | 2 | |  | Booni | | Khyber Pakhtunkhawa | |
| S28 | 1732 | | 0 | | 0 | | 0 | | Sheep | | 3 | |  | Booni | | Khyber Pakhtunkhawa | |
| S29 | 1776 | | 0 | | 0 | | 0 | | Sheep | | 4 | |  | Booni | | Khyber Pakhtunkhawa | |
| S30 | 1139 | | 0 | | 0 | | 0 | | Sheep | | 5 | |  | Booni | | Khyber Pakhtunkhawa | |
| S31 | 1108 | | 0 | | 0 | | 0 | | Sheep | | 6 | |  | Booni | | Khyber Pakhtunkhawa | |
| S32 | 1147 | | 0 | | 0 | | 0 | | Sheep | | 7 | |  | Booni | | Khyber Pakhtunkhawa | |
| S33 | 1956 | | 0 | | 0 | | 0 | | Sheep | | 8 | |  | Booni | | Khyber Pakhtunkhawa | |
| S34 | 1524 | | 0 | | 0 | | 0 | | Sheep | | 9 | |  | Booni | | Khyber Pakhtunkhawa | |
| S35 | 1905 | | 0 | | 0 | | 0 | | Sheep | | 10 | |  | Booni | | Khyber Pakhtunkhawa | |
| S36 | 1233 | | 0 | | 0 | | 0 | | Sheep | | 11 | |  | Booni | | Khyber Pakhtunkhawa | |
| S37 | 1883 | | 0 | | 0 | | 0 | | Sheep | | 12 | |  | Booni | | Khyber Pakhtunkhawa | |
| S38 | 1218 | | 0 | | 0 | | 0 | | Sheep | | 1 | | P 4 | Torkhow | | Khyber Pakhtunkhawa | |
| S39 | 1116 | | 0 | | 0 | | 0 | | Sheep | | 2 | |  | Torkhow | | Khyber Pakhtunkhawa | |
| S40 | 1270 | | 0 | | 0 | | 0 | | Sheep | | 3 | |  | Torkhow | | Khyber Pakhtunkhawa | |
| S41 | 1451 | | 0 | | 0 | | 0 | | Sheep | | 4 | |  | Torkhow | | Khyber Pakhtunkhawa | |
| S42 | 1353 | | 0 | | 0 | | 0 | | Sheep | | 5 | |  | Torkhow | | Khyber Pakhtunkhawa | |
| S43 | 1628 | | 0 | | 0 | | 0 | | Sheep | | 6 | |  | Torkhow | | Khyber Pakhtunkhawa | |
| S44 | 1224 | | 0 | | 0 | | 0 | | Sheep | | 7 | |  | Torkhow | | Khyber Pakhtunkhawa | |
| S45 | 1638 | | 0 | | 0 | | 0 | | Sheep | | 8 | |  | Torkhow | | Khyber Pakhtunkhawa | |
| S46 | 1277 | | 0 | | 0 | | 0 | | Sheep | | 9 | |  | Torkhow | | Khyber Pakhtunkhawa | |
| S47 | 1339 | | 0 | | 0 | | 0 | | Sheep | | 10 | |  | Torkhow | | Khyber Pakhtunkhawa | |
| S48 | 1677 | | 0 | | 0 | | 0 | | Sheep | | 11 | |  | Torkhow | | Khyber Pakhtunkhawa | |
| S49 | 1317 | | 0 | | 0 | | 0 | | Sheep | | 12 | |  | Torkhow | | Khyber Pakhtunkhawa | |
| S50 | 1295 | | 0 | | 0 | | 0 | | Sheep | | 1 | | P 5 | Mastuj | | Khyber Pakhtunkhawa | |
| S51 | 1551 | | 0 | | 0 | | 0 | | Sheep | | 2 | |  | Mastuj | | Khyber Pakhtunkhawa | |
| S52 | 1289 | | 0 | | 0 | | 0 | | Sheep | | 3 | |  | Mastuj | | Khyber Pakhtunkhawa | |
| S53 | 1418 | | 0 | | 0 | | 0 | | Sheep | | 4 | |  | Mastuj | | Khyber Pakhtunkhawa | |
| S54 | 1316 | | 0 | | 0 | | 0 | | Sheep | | 5 | |  | Mastuj | | Khyber Pakhtunkhawa | |
| S55 | 1215 | | 0 | | 0 | | 0 | | Sheep | | 6 | |  | Mastuj | | Khyber Pakhtunkhawa | |
| S56 | 1465 | | 0 | | 0 | | 0 | | Sheep | | 7 | |  | Mastuj | | Khyber Pakhtunkhawa | |
| S57 | 1509 | | 0 | | 0 | | 0 | | Sheep | | 8 | |  | Mastuj | | Khyber Pakhtunkhawa | |
| S58 | 1237 | | 0 | | 0 | | 0 | | Sheep | | 9 | |  | Mastuj | | Khyber Pakhtunkhawa | |
| S59 | 1277 | | 0 | | 0 | | 0 | | Sheep | | 10 | |  | Mastuj | | Khyber Pakhtunkhawa | |
| S60 | 1703 | | 0 | | 0 | | 0 | | Sheep | | 11 | |  | Mastuj | | Khyber Pakhtunkhawa | |
| S61 | 1690 | | 0 | | 0 | | 0 | | Sheep | | 12 | |  | Mastuj | | Khyber Pakhtunkhawa | |
| S62 | 1254 | | 0 | | 0 | | 0 | | Sheep | | 13 | |  | Mastuj | | Khyber Pakhtunkhawa | |
| S63 | 1437 | | 0 | | 0 | | 0 | | Sheep | | 1 | | P 6 | Laspoor valley | | Khyber Pakhtunkhawa | |
| S64 | 1624 | | 0 | | 0 | | 0 | | Sheep | | 2 | |  | Laspoor valley | | Khyber Pakhtunkhawa | |
| S65 | 1434 | | 0 | | 0 | | 0 | | Sheep | | 3 | |  | Laspoor valley | | Khyber Pakhtunkhawa | |
| S66 | 1127 | | 0 | | 0 | | 0 | | Sheep | | 4 | |  | Laspoor valley | | Khyber Pakhtunkhawa | |
| S67 | 1262 | | 0 | | 0 | | 0 | | Sheep | | 5 | |  | Laspoor valley | | Khyber Pakhtunkhawa | |
| S68 | 1895 | | 0 | | 0 | | 0 | | Sheep | | 6 | |  | Laspoor valley | | Khyber Pakhtunkhawa | |
| S69 | 1243 | | 0 | | 0 | | 0 | | Sheep | | 7 | |  | Laspoor valley | | Khyber Pakhtunkhawa | |
| S70 | 1604 | | 0 | | 0 | | 0 | | Sheep | | 8 | |  | Laspoor valley | | Khyber Pakhtunkhawa | |
| S71 | 1232 | | 0 | | 0 | | 0 | | Sheep | | 9 | |  | Laspoor valley | | Khyber Pakhtunkhawa | |
| S72 | 1110 | | 0 | | 0 | | 0 | | Sheep | | 10 | |  | Laspoor valley | | Khyber Pakhtunkhawa | |
| S73 | 1386 | | 0 | | 0 | | 0 | | Sheep | | 11 | |  | Laspoor valley | | Khyber Pakhtunkhawa | |
| S74 | 1760 | | 0 | | 0 | | 0 | | Sheep | | 12 | |  | Laspoor valley | | Khyber Pakhtunkhawa | |
| S75 | 1919 | | 0 | | 0 | | 0 | | Sheep | | 1 | | P 7 | Brun | | Khyber Pakhtunkhawa | |
| S76 | 1534 | | 0 | | 0 | | 0 | | Sheep | | 2 | |  | Brun | | Khyber Pakhtunkhawa | |
| S77 | 1451 | | 0 | | 0 | | 0 | | Sheep | | 3 | |  | Brun | | Khyber Pakhtunkhawa | |
| S78 | 2175 | | 0 | | 0 | | 0 | | Sheep | | 4 | |  | Brun | | Khyber Pakhtunkhawa | |
| S79 | 1044 | | 0 | | 0 | | 0 | | Sheep | | 5 | |  | Brun | | Khyber Pakhtunkhawa | |
| S80 | 1520 | | 0 | | 0 | | 0 | | Sheep | | 6 | |  | Brun | | Khyber Pakhtunkhawa | |
| S81 | 1592 | | 0 | | 0 | | 0 | | Sheep | | 7 | |  | Brun | | Khyber Pakhtunkhawa | |
| S82 | 1359 | | 0 | | 0 | | 0 | | Sheep | | 8 | |  | Brun | | Khyber Pakhtunkhawa | |
| S83 | 1509 | | 0 | | 0 | | 0 | | Sheep | | 9 | |  | Brun | | Khyber Pakhtunkhawa | |
| S84 | 1811 | | 0 | | 0 | | 0 | | Sheep | | 10 | |  | Brun | | Khyber Pakhtunkhawa | |
| S85 | 1298 | | 0 | | 0 | | 0 | | Sheep | | 11 | |  | Brun | | Khyber Pakhtunkhawa | |
| S86 | 1466 | | 0 | | 0 | | 0 | | Sheep | | 12 | |  | Brun | | Khyber Pakhtunkhawa | |
| S87 | 1527 | | 0 | | 0 | | 0 | | Sheep | | 1 | | P 8 | Dalomal | | Gilgit Baltistan | |
| S88 | 1245 | | 0 | | 0 | | 0 | | Sheep | | 2 | |  | Dalomal | | Gilgit Baltistan | |
| S89 | 1371 | | 0 | | 0 | | 0 | | Sheep | | 3 | |  | Dalomal | | Gilgit Baltistan | |
| S90 | 1205 | | 0 | | 0 | | 0 | | Sheep | | 4 | |  | Dalomal | | Gilgit Baltistan | |
| S91 | 1202 | | 0 | | 0 | | 0 | | Sheep | | 5 | |  | Dalomal | | Gilgit Baltistan | |
| S92 | 1343 | | 0 | | 0 | | 0 | | Sheep | | 6 | |  | Dalomal | | Gilgit Baltistan | |
| S93 | 1402 | | 0 | | 0 | | 0 | | Sheep | | 7 | |  | Dalomal | | Gilgit Baltistan | |
| S94 | 1324 | | 0 | | 0 | | 0 | | Sheep | | 8 | |  | Dalomal | | Gilgit Baltistan | |
| S95 | 1112 | | 0 | | 0 | | 0 | | Sheep | | 9 | |  | Dalomal | | Gilgit Baltistan | |
| S96 | 1224 | | 0 | | 0 | | 0 | | Sheep | | 10 | |  | Dalomal | | Gilgit Baltistan | |
| S97 | 2104 | | 0 | | 0 | | 0 | | Sheep | | 11 | |  | Dalomal | | Gilgit Baltistan | |
| S98 | 1406 | | 0 | | 0 | | 0 | | Sheep | | 12 | |  | Dalomal | | Gilgit Baltistan | |
| S99 | 1792 | | 0 | | 0 | | 0 | | Sheep | | 1 | | P 9 | Yasin Valley | | Gilgit Baltistan | |
| S100 | 1033 | | 0 | | 0 | | 0 | | Sheep | | 2 | |  | Yasin Valley | | Gilgit Baltistan | |
| S101 | 1680 | | 0 | | 0 | | 0 | | Sheep | | 3 | |  | Yasin Valley | | Gilgit Baltistan | |
| S102 | 2591 | | 0 | | 0 | | 0 | | Sheep | | 4 | |  | Yasin Valley | | Gilgit Baltistan | |
| S103 | 1852 | | 0 | | 0 | | 0 | | Sheep | | 5 | |  | Yasin Valley | | Gilgit Baltistan | |
| S104 | 2101 | | 0 | | 0 | | 0 | | Sheep | | 6 | |  | Yasin Valley | | Gilgit Baltistan | |
| S105 | 2046 | | 0 | | 0 | | 0 | | Sheep | | 7 | |  | Yasin Valley | | Gilgit Baltistan | |
| S106 | 1523 | | 0 | | 0 | | 0 | | Sheep | | 8 | |  | Yasin Valley | | Gilgit Baltistan | |
| S107 | 2220 | | 0 | | 0 | | 0 | | Sheep | | 9 | |  | Yasin Valley | | Gilgit Baltistan | |
| S108 | 1534 | | 0 | | 0 | | 0 | | Sheep | | 10 | |  | Yasin Valley | | Gilgit Baltistan | |
| S109 | 2182 | | 0 | | 0 | | 0 | | Sheep | | 11 | |  | Yasin Valley | | Gilgit Baltistan | |
| S110 | 2837 | | 0 | | 0 | | 0 | | Sheep | | 12 | |  | Yasin Valley | | Gilgit Baltistan | |
| S111 | 1236 | | 0 | | 0 | | 0 | | Sheep | | 1 | | P 10 | Raushan | | Gilgit Baltistan | |
| S112 | 1549 | | 0 | | 0 | | 0 | | Sheep | | 2 | |  | Raushan | | Gilgit Baltistan | |
| S113 | 1310 | | 0 | | 0 | | 0 | | Sheep | | 3 | |  | Raushan | | Gilgit Baltistan | |
| S114 | 2292 | | 0 | | 0 | | 0 | | Sheep | | 4 | |  | Raushan | | Gilgit Baltistan | |
| S115 | 1420 | | 0 | | 0 | | 0 | | Sheep | | 5 | |  | Raushan | | Gilgit Baltistan | |
| S116 | 2392 | | 0 | | 0 | | 0 | | Sheep | | 6 | |  | Raushan | | Gilgit Baltistan | |
| S117 | 1119 | | 0 | | 0 | | 0 | | Sheep | | 7 | |  | Raushan | | Gilgit Baltistan | |
| S118 | 1584 | | 0 | | 0 | | 0 | | Sheep | | 8 | |  | Raushan | | Gilgit Baltistan | |
| S119 | 2470 | | 0 | | 0 | | 0 | | Sheep | | 9 | |  | Raushan | | Gilgit Baltistan | |
| S120 | 2191 | | 0 | | 0 | | 0 | | Sheep | | 10 | |  | Raushan | | Gilgit Baltistan | |
| S121 | 1544 | | 0 | | 0 | | 0 | | Sheep | | 11 | |  | Raushan | | Gilgit Baltistan | |
| S122 | 2865 | | 0 | | 0 | | 0 | | Sheep | | 12 | |  | Raushan | | Gilgit Baltistan | |
| S123 | 1003 | | 0 | | 0 | | 0 | | Sheep | | 1 | | P 11 | Raushan | | Gilgit Baltistan | |
| S124 | 1184 | | 0 | | 0 | | 0 | | Sheep | | 2 | |  | Raushan | | Gilgit Baltistan | |
| S125 | 3216 | | 0 | | 0 | | 0 | | Sheep | | 3 | |  | Raushan | | Gilgit Baltistan | |
| S126 | 2940 | | 0 | | 0 | | 0 | | Sheep | | 4 | |  | Raushan | | Gilgit Baltistan | |
| S127 | 2184 | | 0 | | 0 | | 0 | | Sheep | | 5 | |  | Raushan | | Gilgit Baltistan | |
| S128 | 2610 | | 0 | | 0 | | 0 | | Sheep | | 6 | |  | Raushan | | Gilgit Baltistan | |
| S129 | 2142 | | 0 | | 0 | | 0 | | Sheep | | 7 | |  | Raushan | | Gilgit Baltistan | |
| S130 | 1634 | | 0 | | 0 | | 0 | | Sheep | | 8 | |  | Raushan | | Gilgit Baltistan | |
| S131 | 3345 | | 0 | | 0 | | 0 | | Sheep | | 9 | |  | Raushan | | Gilgit Baltistan | |
| S132 | 2950 | | 0 | | 0 | | 0 | | Sheep | | 10 | |  | Raushan | | Gilgit Baltistan | |
| S133 | 1697 | | 0 | | 0 | | 0 | | Sheep | | 1 | | P 12 | Gabral | | Khyber Pakhtunkhawa | |
| S134 | 2518 | | 0 | | 0 | | 0 | | Sheep | | 2 | |  | Gabral | | Khyber Pakhtunkhawa | |
| S135 | 1847 | | 0 | | 0 | | 0 | | Sheep | | 3 | |  | Gabral | | Khyber Pakhtunkhawa | |
| S136 | 508 | | 0 | | 0 | | 0 | | Sheep | | 4 | |  | Gabral | | Khyber Pakhtunkhawa | |
| S137 | 1329 | | 0 | | 0 | | 0 | | Sheep | | 5 | |  | Gabral | | Khyber Pakhtunkhawa | |
| S138 | 3044 | | 0 | | 0 | | 0 | | Sheep | | 6 | |  | Gabral | | Khyber Pakhtunkhawa | |
| S139 | 2350 | | 0 | | 0 | | 0 | | Sheep | | 7 | |  | Gabral | | Khyber Pakhtunkhawa | |
| S140 | 1810 | | 0 | | 0 | | 0 | | Sheep | | 8 | |  | Gabral | | Khyber Pakhtunkhawa | |
| S141 | 1694 | | 0 | | 0 | | 0 | | Sheep | | 9 | |  | Gabral | | Khyber Pakhtunkhawa | |
| S142 | 1397 | | 0 | | 0 | | 0 | | Sheep | | 10 | |  | Gabral | | Khyber Pakhtunkhawa | |
| S143 | 2705 | | 0 | | 0 | | 0 | | Sheep | | 11 | |  | Gabral | | Khyber Pakhtunkhawa | |
| S144 | 2004 | | 0 | | 0 | | 0 | | Sheep | | 12 | |  | Gabral | | Khyber Pakhtunkhawa | |
| S145 | 1297 | | 0 | | 0 | | 0 | | Sheep | | 1 | | P 13 | Boyun | | Khyber Pakhtunkhawa | |
| S146 | 2503 | | 0 | | 0 | | 0 | | Sheep | | 2 | |  | Boyun | | Khyber Pakhtunkhawa | |
| S147 | 1313 | | 0 | | 0 | | 0 | | Sheep | | 3 | |  | Boyun | | Khyber Pakhtunkhawa | |
| S148 | 1796 | | 0 | | 0 | | 0 | | Sheep | | 4 | |  | Boyun | | Khyber Pakhtunkhawa | |
| S149 | 1081 | | 0 | | 0 | | 0 | | Sheep | | 5 | |  | Boyun | | Khyber Pakhtunkhawa | |
| S150 | 2968 | | 0 | | 0 | | 0 | | Sheep | | 6 | |  | Boyun | | Khyber Pakhtunkhawa | |
| S151 | 1423 | | 0 | | 0 | | 0 | | Sheep | | 7 | |  | Boyun | | Khyber Pakhtunkhawa | |
| S152 | 2239 | | 0 | | 0 | | 0 | | Sheep | | 8 | |  | Boyun | | Khyber Pakhtunkhawa | |
| S153 | 0 | | 0 | | 0 | | 0 | | Sheep | | 9 | |  | Boyun | | Khyber Pakhtunkhawa | |
| S154 | 1540 | | 0 | | 0 | | 0 | | Sheep | | 10 | |  | Boyun | | Khyber Pakhtunkhawa | |
| S155 | 2538 | | 0 | | 0 | | 0 | | Sheep | | 11 | |  | Boyun | | Khyber Pakhtunkhawa | |
| S156 | 2310 | | 0 | | 0 | | 0 | | Sheep | | 12 | |  | Boyun | | Khyber Pakhtunkhawa | |
| S157 | 1669 | | 0 | | 0 | | 0 | | Goat | | 1 | | P 14 | Chinar | | Khyber Pakhtunkhawa | |
| S158 | 1400 | | 0 | | 0 | | 0 | | Goat | | 2 | |  | Chinar | | Khyber Pakhtunkhawa | |
| S159 | 162 | | 0 | | 0 | | 0 | | Goat | | 3 | |  | Chinar | | Khyber Pakhtunkhawa | |
| S160 | 1108 | | 0 | | 0 | | 0 | | Goat | | 4 | |  | Chinar | | Khyber Pakhtunkhawa | |
| S161 | 2190 | | 0 | | 0 | | 0 | | Goat | | 5 | |  | Chinar | | Khyber Pakhtunkhawa | |
| S162 | 3642 | | 0 | | 0 | | 0 | | Goat | | 6 | |  | Chinar | | Khyber Pakhtunkhawa | |
| S163 | 1424 | | 0 | | 0 | | 0 | | Goat | | 7 | |  | Chinar | | Khyber Pakhtunkhawa | |
| S164 | 2131 | | 0 | | 0 | | 0 | | Goat | | 8 | |  | Chinar | | Khyber Pakhtunkhawa | |
| S165 | 2239 | | 0 | | 0 | | 0 | | Goat | | 9 | |  | Chinar | | Khyber Pakhtunkhawa | |
| S166 | 2306 | | 0 | | 0 | | 0 | | Goat | | 10 | |  | Chinar | | Khyber Pakhtunkhawa | |
| S167 | 3103 | | 0 | | 0 | | 0 | | Goat | | 11 | |  | Chinar | | Khyber Pakhtunkhawa | |
| S168 | 2817 | | 0 | | 0 | | 0 | | Goat | | 12 | |  | Chinar | | Khyber Pakhtunkhawa | |
| S169 | 2339 | | 0 | | 0 | | 0 | | Goat | | 1 | | P 15 | Gasht | | Khyber Pakhtunkhawa | |
| S170 | 1966 | | 0 | | 0 | | 0 | | Goat | | 2 | |  | Gasht | | Khyber Pakhtunkhawa | |
| S171 | 2054 | | 0 | | 0 | | 0 | | Goat | | 3 | |  | Gasht | | Khyber Pakhtunkhawa | |
| S172 | 1886 | | 0 | | 0 | | 0 | | Goat | | 4 | |  | Gasht | | Khyber Pakhtunkhawa | |
| S173 | 2302 | | 0 | | 0 | | 0 | | Goat | | 5 | |  | Gasht | | Khyber Pakhtunkhawa | |
| S174 | 2274 | | 0 | | 0 | | 0 | | Goat | | 6 | |  | Gasht | | Khyber Pakhtunkhawa | |
| S175 | 1749 | | 0 | | 0 | | 0 | | Goat | | 7 | |  | Gasht | | Khyber Pakhtunkhawa | |
| S176 | 2392 | | 0 | | 0 | | 0 | | Goat | | 8 | |  | Gasht | | Khyber Pakhtunkhawa | |
| S177 | 1236 | | 0 | | 0 | | 0 | | Goat | | 9 | |  | Gasht | | Khyber Pakhtunkhawa | |
| S178 | 2055 | | 0 | | 0 | | 0 | | Goat | | 10 | |  | Gasht | | Khyber Pakhtunkhawa | |
| S179 | 2717 | | 0 | | 0 | | 0 | | Goat | | 11 | |  | Gasht | | Khyber Pakhtunkhawa | |
| S180 | 2246 | | 0 | | 0 | | 0 | | Goat | | 12 | |  | Gasht | | Khyber Pakhtunkhawa | |
| S181 | 1726 | | 0 | | 0 | | 0 | | Goat | | 1 | | P 16 | Chalt Nagar | | Gilgit Baltistan | |
| S182 | 1359 | | 0 | | 0 | | 0 | | Goat | | 2 | |  | Chalt Nagar | | Gilgit Baltistan | |
| S183 | 0 | | 0 | | 0 | | 0 | | Goat | | 3 | |  | Chalt Nagar | | Gilgit Baltistan | |
| S184 | 0 | | 0 | | 0 | | 0 | | Goat | | 4 | |  | Chalt Nagar | | Gilgit Baltistan | |
| S185 | 1411 | | 0 | | 0 | | 0 | | Goat | | 5 | |  | Chalt Nagar | | Gilgit Baltistan | |
| S186 | 1763 | | 0 | | 0 | | 0 | | Goat | | 6 | |  | Chalt Nagar | | Gilgit Baltistan | |
| S187 | 1519 | | 0 | | 0 | | 0 | | Goat | | 7 | |  | Chalt Nagar | | Gilgit Baltistan | |
| S188 | 0 | | 0 | | 0 | | 0 | | Goat | | 8 | |  | Chalt Nagar | | Gilgit Baltistan | |
| S189 | 1615 | | 0 | | 0 | | 0 | | Goat | | 9 | |  | Chalt Nagar | | Gilgit Baltistan | |
| S190 | 1541 | | 0 | | 0 | | 0 | | Goat | | 10 | |  | Chalt Nagar | | Gilgit Baltistan | |
| S191 | 1361 | | 0 | | 0 | | 0 | | Goat | | 11 | |  | Chalt Nagar | | Gilgit Baltistan | |
| S192 | 1324 | | 0 | | 0 | | 0 | | Goat | | 912 | |  | Chalt Nagar | | Gilgit Baltistan | |
